# Assessment of Connective Tissue in the Equine Uterus and Cervix: Review of Clinical Impact and Staining Options

**DOI:** 10.3390/ani14010156

**Published:** 2024-01-03

**Authors:** Łukasz Zdrojkowski, Bartosz Pawliński, Katarzyna Skierbiszewska, Tomasz Jasiński, Małgorzata Domino

**Affiliations:** Department of Large Animal Diseases and Clinic, Institute of Veterinary Medicine, Warsaw University of Life Sciences (WULS–SGGW), 02-787 Warsaw, Poland; bartosz_pawlinski@sggw.edu.pl (B.P.); katarzyna_skierbiszewska@sggw.edu.pl (K.S.); tomasz_jasinski@sggw.edu.pl (T.J.)

**Keywords:** fibrosis, angiosis, endometrosis, connective tissue, cervix, infertility, mare

## Abstract

**Simple Summary:**

Uterine diseases are the leading cause of infertility in mares, causing increasing costs and losses in horses’ breeding. Their current diagnosis is often supported by obtaining endometrial biopsies and hematoxylin–eosin staining, which is the basic staining used in histopathology. This review aims to show the variety of uterine changes affecting fertility and highlights the usefulness of special stains for connective tissue visualization—Masson trichrome, picrosirius red, elastica van Gieson, or periodic acid–Schiff—for a more comprehensive diagnosis. The fibrosis evaluation includes connective tissue changes in the cervix, the endometrium, and around/in the wall of blood vessels. Cervical connective tissue undergoes cyclic changes impacting fertility, whereas vascular changes, especially in multiparous mares, are crucial for adapting to physiological shifts, affecting early pregnancy and hindering placental development. Special stains are valuable for the identification of structural changes in the cervix and fibrosis in uterine blood vessels. Moreover, equine endometrosis, linked to fibrotic processes in the endometrium, emphasizes the need for wider use of special stains in diagnosis. Therefore, we advocate for special staining in reproductive tract evaluation due to its simplicity, accessibility, and effectiveness. We encourage scientists and diagnosticians to adopt additional tools for clearer pathology visualization, enabling reliable fertility predictions.

**Abstract:**

Uterine diseases stand as the primary cause of infertility in mares; however, the diagnostic process often relies on obtaining endometrial biopsies and their hematoxylin–eosin staining. This review seeks to present the variability of uterine changes and their impact on fertility and underscore the utility of special stains, such as Masson trichrome, picrosirius red, elastica van Gieson, or periodic acid–Schiff, in enhancing diagnostic breadth. Connective tissue evaluation in the cervix is discussed, as it is subjected to cyclic changes and the impact on overall fertility. Vascular changes, particularly prevalent in multiparous mares, play a crucial role in adapting to physiological and pathological alterations, affecting early gestation and impeding placental development. Given that uterine vascular pathologies often involve fibrotic changes, connective tissue stains emerge as a valuable tool in this context. Moreover, equine endometriosis, predominantly associated with endometrial fibrosis, further highlights the relevance of special stains, suggesting their underutilization in the diagnostic process. Recognizing the subjective nature of diagnosing uterine pathologies and the need for additional diagnostic tools, we advocate for using dedicated stains in the histopathological evaluation of uterine samples. In conclusion, we encourage scientists and diagnosticians to embrace additional tools that enhance pathology visualization, enabling more reliable diagnoses concerning expected fertility.

## 1. Fibrosis Formation and Its Staining Possibilities in the Equine Uterus

Infertility is considered a major health problem in mares, as horses, among other domestic animals, have the lowest fertility rates, which is associated with their longevity and usage [1,2,3]. Age-related uterine diseases, such as endometritis, endometrosis, glandular differentiation disorders, and angiosis [3,4,5,6], are especially important, as improved management and care elongate lifespan, and many mares are bred only after successful sport or racing career [1,2]. Thus, especially in horses, a thorough and reliable diagnosis regarding fertility prognosis seems to be an important issue affecting the profitability of horse breeding. Sub- or infertility is causing economic losses in the equine industry and hampering efficient and effective breeding, as well as genotypic improvements among breeds [1,7]. 

Endometritis is associated with an altered uterine environment and infertility [2,3]. In the first 72 h after breeding, endometritis is the physiological inflammatory reaction in the endometrium [8,9,10]. If inflammation persists beyond this timeframe, for example, due to impaired uterine clearance, it converts into persistent endometritis, while an embryo dies [8,10,11]. On the other hand, the term “endometrosis”, initially introduced by Kenney [12,13] and modified by Schoon et al. [11], defines the active or inactive periglandular and/or stromal endometrial fibrosis, including glandular alterations within fibrotic foci and destructive or nondestructive changes in the glandular epithelium [14]. The major diagnostic features include the proportion of affected glands and the layers of collagen fibers enclosing a gland, inflammation, and reported infertility [4,15,16,17,18], which were proposed in the Kenney–Doig classification scheme for rating endometritis and endometrosis histological changes as absent (I), mild (IIA), moderate (IIB), or severe (III) [12]. In recent research on this chronic disease [5,12,14,19], the terms “degenerative endometrial fibrosis” [20] or “endometrial degeneration” [21] are used interchangeably. Regardless of the nomenclature, it involves periglandular fibrosis, altering the stromal structure and resulting in cystic glandular dilation [12,14,17,22]. Single-gland branched structures and/or multiple glands can be affected, with the latter often referred to as glandular nests or “nested endometrosis” [3]. In severe cases, multifocal–diffuse fibrosis may be observed [23]. The pathological alterations of the endometrial glands are associated with abnormal glandular function [14,24], which might contribute to estrous cycle dysregulation and early embryonic loss [25]. Endometrial fibrosis is often linked to concurrent endometritis [2,14,26]. Therefore, both are considered in the Kenney–Doig classification scheme and collectively impact the severity of histological changes in the equine endometrium as reversible and irreversible alterations [12,18,27]. While both may occur independently, endometrosis is considered a risk factor for endometritis, as local immunity mechanisms may not be sufficient, whether because of endometrial degeneration or age-associated changes [3,28]. Therefore, equine endometrosis is a common fertility-reducing disease of the equine endometrium [3], with the etiology and pathogenesis remaining unclear [3,14,28,29]. 

Glandular differentiation disorders may manifest during the breeding season as irregular glandular differentiation and unequal glandular differentiation [3,6,30]. The former can be present as irregular secretory, irregular proliferative, or complete irregular differentiation, while the latter is characterized by focal or multifocal groups of glands with asynchronous differentiation throughout the cycle [31,32]. In both cases, abnormal expression of hormone receptors [31,32,33], altered protein secretion [34], and disruption of the basement membrane of glandular epithelial cells have been observed [32]. However, no periglandular fibrosis was noted in these cases [3]. 

Finally, angiosis is a common angiopathy which occurs in the equine endometrium [35]. In recent research on this degenerative disease, the term “angiosclerosis” has been used interchangeably [35]. Angiosis does not encompass all abnormalities of blood vessels. In contrast to less common inflammatory lesions like perivasculitis and vasculitis, angiosis is characterized by an augmented deposition of connective tissue and/or elastic fibers within the wall of endometrial vessels [35,36,37].

It is evident that two out of the four primary endometrial diseases, which are the main causes of sub- and infertility [3,15,17,18,35,37,38,39,40], are associated with the formation of fibrosis and thus the deposition of connective tissue in the equine endometrium. The histopathological examination of endometrial biopsy or full-thickness sections remains indispensable in assessing the alterations in the uterine wall structure [3,13,39,40]; hematoxylin and eosin (HE) is a routinely used staining method to visualize tissue morphology. The HE stain is commonly used to assess connective tissue fibers as well, besides the availability of well-established histochemical methods for the visualization of connective tissue and fibrosis [1,5,41,42,43,44,45,46,47]. As the HE is not specific to connective tissue, the use of special stains is potentially beneficial, especially for the quantitative assessment of fibrosis. Although the histological-specific staining methods are inexpensive, easy to perform, and informative, the usability of specific connective tissue stains in endometrium assessment is often questioned, and despite recurrent use in research, its application in routine clinical diagnosis is rather limited [3,5,15,22,27,35,43,48]. In research, sometimes only the application of special staining is mentioned, suggesting that the purpose is to facilitate fibrosis assessment [22,48,49]. Among the specific staining for connective tissue, Masson trichrome staining (MT) [43,50,51,52,53,54,55,56,57], picrosirius red staining (PSR) [15,18,35,58,59,60], Gomori trichrome staining (GT) [61,62], elastica van Gieson staining (EVG) [4,63,64], Azan staining [65], resorcin–fuchsin staining (RF) [65], periodic acid–Schiff staining (PAS) [4,52,65,66], and, in highly specific way, IHC [6,16,51,60,67], may be utilized for enhancing the histological visualization of connective tissue in light microscopy. Although electron microscopy is a more expensive technique, it may be employed for the evaluation of smaller structures in ECM or cellular degeneration in fibrosis studies; thus, methylene-blue-azur2-basic fuchsin staining (MBABF) on semi-thin slices for area selection [35], uranyl acetate–lead citrate staining (UALC) [4,35,64], and gold-coating [64,66] can be used. While some claim that specific staining does not provide any additional value to the breeding soundness exam [15,43], assessing the scale and severity of fibrosis is considered more reliable when a special stain is used. Moreover, in scientific research on the assessment of connective tissue in the endometrium, the specific staining for fibers is widely used and recommended [45,46,47,68,69,70,71]. This review serves to systematically summarize the usefulness of methods of connective tissue staining in the equine uterus, which make the diagnosis of endometrial and cervical diseases more accurate. The accurate diagnosis, which is the result of cooperation between clinicians and diagnosticians, enables owners to make informed decisions about further breeding, taking into consideration not only the numeric categorization of endometrial biopsy but also the qualitative and quantitative assessment of each individual case [1,72].

This review aims to discuss methods for connective tissue staining in the uterus and cervix, as well as their role in the assessment of diseases associated with fibrosis in the context of the mares’ fertility.

## 2. Measurement and Influence of Fibrotic Processes

This critical review is based on a search of articles in English in PubMed http://www.ncbi.nlm.nih.gov/pubmed (accessed on 30 October 2023) and Scopus https://www.scopus.com/home (accessed on 30 October 2023) using the terms “connective tissue, collagen, fibrosis” combined with localization, i.e., uterus, uterine, endometrium; and species, i.e., mare, equine, since 1986, the year of introduction of endometrium grading method [12]. Libraries were screened to the consecutive page after the last relevant record. Duplicates have been removed. Title and abstract databases of the obtained articles were created, and manuscripts referring to the connective tissue evaluation in a uterus were studied in detail. Studies in which the HE stain was used only for the Kenney–Doig category, without evaluation of fibrosis or its impact on fertility, were not investigated thoroughly. We also did not investigate research on endometrosis pathogenesis, as this area is rapidly developing, and several remarkable review articles have been published recently. 

### 2.1. Assessment of Cervical Functionality

Understanding of cervical cyclic changes may be broadened with histological examination, including specific staining for connective tissue. All research conducted on connective tissue in the equine cervix has investigated the full-thickness post-mortem sections [50,51,65]. A cervical biopsy has the potential to disrupt its structure, causing permanent damage and, consequently, infertility. Therefore, this procedure is not routinely performed, and any manipulation of the cervix outside of estrus is conducted with utmost care [73]. The cervical contraction–relaxation process is dependent on the functioning of active structures (smooth muscle cells) as well as passive structures (connective tissue in the muscular and mucosal layers of the cervical wall). Although the cervix has a lower number of steroid hormones receptors, it is still influenced by the ovarian cycle [33,74]. Hydration of connective tissue increases in the reproductive tract during the follicular phase, which can be seen as edema [3,17,75]. Therefore, an increase in the area covered by connective tissue in histological specimens is more closely associated with connective tissue hydration rather than increased connective tissue synthesis [18]. Reports on the assessment of the connective tissue in the equine cervix are summarized in Table 1.

HE was performed in almost all cervical connective tissue studies [50,65,73]. Thanks to this, the structure of the cervical wall and cell morphology were assessed both in the normal cervix [65,73] as well as in the case of cervicitis [73]. Among the specific staining for connective tissue in the equine cervix, PAS, Azan staining, RF [65], and MT [50,51] were used. The first three stains allowed for visualization of ciliated cells in luminal epithelium and vascularization in the deep lamina propria of mucosae [65], whereas the last was used to assess the cyclic changes in connective tissue in the cervical mucosal and muscular layers [50,51]. During the follicular phase, the share of connective tissue in the tunica mucosa increased, primarily due to the formation of more folds during this phase. No differences were found in the muscular layer, suggesting that it is less susceptible to cyclic changes. There were no differences in the width or number of cell layers between the follicular and luteal phases, indicating that functional changes in the cervix may be primarily dependent on the functioning of the mucosal layer (conference report, unpublished findings) [50].

A higher content of connective tissue was observed in diestrus than in estrus [51], which, however, does not allow us to distinguish whether it was due to increased hydration or synthesis [18]. On the other hand, no cyclic changes were found in luminal epithelium, or in vascular density within the tunica mucosa and tunica muscularis [65]. In a study by Campbell et al. [51], the cervical expression of the estrogen receptor α gene was investigated, and higher values in both the luminal epithelium and stroma were reported during estrus than diestrus [51]. While a measurable estrogen influence is observed in the cervical stroma [3,75,76], the effects of increased estrogen levels in estrus on epithelial cells require further research. 

Interestingly, the presence of a hypothetical venous plexus in the cervix may be a subject of future research, especially concerning endometrial vascular changes in multiparous mares, as described in more detail in Section 2.2 [4,35,65].

### 2.2. Assessment of Vascular Changes

Functional changes in the pregnant uterus involve alterations in blood supply and uterine blood vessels, leading to angioses [4,6,36,37,59,77]. The incidence and severity of these structural changes in the blood vessel walls increase with age and the number of foalings [12,78,79,80]. Angioses are frequently observed as co-occurring with endometrosis [4,37,46], so fibrosis occurs simultaneously in more than one endometrial structure. While the consequences of both types of fibrotic changes are not fully understood [3,6,18,28,29], they should be considered when assessing endometrial specimens. 

Angioses may also reduce the adaptive capacity of vascularization to respond to alterations in uterine blood demand in specific situations [59,77]. The biggest challenge for uterine vascularization is gestation. In the initial stage, when an embryo is still migrating through the uterine lumen, the blood flow in an altered vesselis less associated with the position of the embryonic vesicle [77]. Vascular fibrosis results in a lower density of chorionic villi in the placenta, possibly leading to poorer fetal nutrition [37,59,77]. Additionally, a decrease in uterine blood supply may exacerbate placental development insufficiency. On the other hand, multiparous mares tend to develop heavier placentas, resulting in higher foal mass [77]. Thus, it may be suspected that foal mass at birth initially increases with parity, but at some point, it starts to decrease as vascular changes progress. While it has not been studied in horses, results from other species suggest that occlusion in uterine blood flow results in decreased uterine contractility [59,81]. This aspect may suggest the role of angioses in delayed uterine clearance and intrauterine fluid accumulation, thus influencing susceptibility [3,74]. Similarly, functional adaptation of blood supply is impaired during the estrus cycle. Angioses diminish the overall uterine perfusion, as well as variability in particular anatomical regions, especially in uterine horns, a region with the physiologically highest perfusion [59,77]. Additionally, uterine angioses are associated with reduced ovarian blood supply, which, in turn, leads to decreased hormonal signaling—a crucial factor in the regulation of the seasonal estrous cycle [37]. The influence of angioses suggests a potential deficiency in vascular adaptation during inflammatory processes. Given that the effectiveness and duration of inflammation depend on increased blood flow and rapid neutrophil infiltration, in both infectious and breeding-induced endometritis, it can be hypothesized that angiosis may also prolong or disrupt the healing process, despite the lack of scientific data [81,82]. To facilitate the assessment of histological results, reports on the assessment of the perivascular connective tissue in the equine endometrium are summarized in Table 2.

HE was utilized for the vascular assessment in both biopsy [27,35,40,58,63,77] and full-thickness specimens [4,53,54,55,58,63]. This basic staining was sufficient to evidence that incidence and degree of angiosis increase with mare age [40] with no effect on the uterine blood flow increase in the gestation [77]. Moreover, angioses in arteries result in increased vascular resistance; the reduction of uterine blood supply in pregnancy was not observed [77]. On the other hand, degeneration of veins may lead to blood stasis, hindering the lymphatic drainage and evacuation of intrauterine fluid and potentially exposing the mare to persistent endometritis [4,36,77]. However, again, there were no such observations during pregnancy [77].

Among the specific staining for connective tissue in the case of equine endometrial angiosis, PSR [27,35,58], EVG [4,63], PAS [4], and MT [53,54,55] were utilized. Of them, PSR was recommended as an excellent method for a concise characterization of vascular alterations [59,80]. PSR (light microscopy), MBABF, and UALC (electron microscopy) allowed for correlating the age and parity of a mare with the incidence and degree of angiosis [35]. EVG, PAS (light microscopy), and UALC (electron microscopy) were used in a study demonstrating that incidence and severity begin after the mare’s sixth year of life [4]. The last one was used for the separation of collagen fibers encircling the vessel from the rest of the connective tissue, making intramural connective tissue fibers distinguishable within the vessel wall [53,54,55].

In a preliminary study, angioses associated with endometrosis and endometritis were assessed (conference reports, unpublished findings) [53,54,55]. It was observed that the average vessel wall width in endometritis was significantly reduced in fibrotic samples, which may suggest stretching of the wall caused by blood stasis. On the other hand, the average vessel lumen area was also smaller in endometrosis, likely linked to decreased perfusion [53]. Concerning endometrial biopsies categories, perivascular fibrosis was higher in category III than I, with no differences between them and categories IIA and IIB [54]. It may suggest that angioses, evidenced by perivascular fibrosis, thickened walls of vessels, and increased vessel lumen area, occur more often at the end of the fibrotic degeneration process. The area of perivascular fibrosis was also evaluated in relation to the hyaluronan synthases (HAS) 1, 2, and 3 expressions, showing that perivascular fibrosis may be mediated by HAS 1 and 3 but only in more severe endometrosis [55].

Although angioses are mostly found in multiparous mares, mild changes are also observed in maiden mares [3,36,59,77]. While their uterine vessels were not subjected to deep remodeling during pregnancy, they were still subjected to cyclic and inflammatory vascular changes [3,36,77,82]. Thus, it can be hypothesized that the capability of withstanding continuous adaptations of blood vessels is limited, and the frequency and severity of functional events hasten their loss of functionality. However, individual variability in this feature is significant, as demonstrated by old, multiparous, and still-fertile mares [3,4,22,40,77] with marked vascular changes, while glands remain functional [3,4,40].

### 2.3. Assessment of Endometrial Changes

Although conventional HE is mostly used for histological diagnosis, specific staining for connective tissue appears to be underappreciated in the context of endometrosis, with some suggesting that it does not provide additional value to HE stain [4,15,16,18,43,44]. In contrast, Grüninger et al. stated that MT is widely used for detecting endometrial fibrosis [15]. Interestingly, in some scientific studies, mostly MT [16,22,43,52,56,57] and PSR [15,18,60] were utilized to stain collagen fibers, while EVG [4,64], PAS [4,52,66], Alcian blue (AB) staining [16,52], immunohistochemical (IHC) staining [6,16,60,67] (light microscopy), UALC [4,64], and gold sputter (GS) coating [66] (electron microscopy) were used for other specific assessments of other ECM components co-occurring alterations. Such a wide use of specific staining in the scientific research indicates that for diagnostic purposes, connective tissue staining would be beneficial. It is worth noting that some studies on connective tissue quantification in relation to the clinical outcome and the expected fertility in the case of endometrosis were already presented [5,15]. 

Visualization of collagen fibers may allow for a better assessment of the density of the connective tissue surrounding endometrial glands. Increased density can be responsible for the impaired transport of nutrients and endocrine signaling molecules from blood vessels to the glandular epithelium; thus, it may cause degeneration and loss of function [83,84]. The specific staining methods offer superior contrast between collagen fibers and glands. Moreover, specific staining may support the distinction between endometrosis and glandular differentiation disorders [5,38,72]. While specific staining has been discussed in relation to periglandular or stromal fibrosis, there is a lack of data regarding the visualization of destructive or nondestructive changes in glandular epithelium. To facilitate the assessment of histopathological results, reports on the assessment of the connective tissue in the equine endometrosis were summarized in Table 3.

Recent preliminary work presented the differences in cytoplasmic staining of glandular epithelium (conference reports, unpublished findings) [56,57]. All samples were stained with HE and assessed in a standard manner, while MT-stained slides were examined using both light microscopy and a scanning cytometer. Initially, lighter cytoplasm was observed in glandular epithelium with destructive changes, while darker cytoplasm was supported with nondestructive glandular epithelium [56]. Fibrotic areas around glands were found to be larger when cytoplasm was marked as light, and no differences were found around glands with dark cytoplasm, suspected to be functional [56]. The occurrence of glands with light cytoplasm was higher in endometrosis than in healthy endometrium [57]. However, further research is needed to evidence whether areas of glands with lighter cytoplasm are related to clinical outcome or fertility prognosis [56,57].

## 3. Comparison of Staining Methods of the Connective Tissue

In previous studies, several methods for connective tissue evaluation were employed. As they use various stains, differently binding connective tissue compounds each provide a specific value; however, each is also associated with some limitations or flaws [85,86]. Thus, staining selection should be directly related to the aim of the study and the possibility of evaluation. HE is globally used for the evaluation of cell morphology and tissue architecture [45]. It is commonly used to diagnose any histological alterations in a tissue, including fibrosis in endometrial samples, although it is not a specific stain for the connective tissue [68,69,87,88]. This implies that the ECM evaluation in HE-stained slides may be biased and result in higher variability in the observer impact [47,71]. Some researchers claim that reticular fibers are not stained in HE, collagen fibers may not be differentiable, and special stains may replace the use of advanced techniques [45,46,68,69,70]. Conversely, some studies showed a lack of benefits coming from using special stains, apart from HE [43,71]. Regarding stains dedicated to connective tissue, MT and PSR are considered relatively easy, fast, and inexpensive stains with high throughput, making them usable in a standard diagnostic process [85,86,89,90,91]. As GT uses a single stain for ECM, it is also easy to perform [47,92]. On the other hand, along with EVG (for elastic fibers), RF, and Azan staining, GT is variable in the differentiation stage, causing equal staining intensity to be harder to achieve [86,89,92,93]. Azan and RF are also time-consuming [93,94].

While MT stains ECM collectively (smaller collagen fibers may not be stained, causing an underestimation of collagen content [88,89]), RF and EVG stains (EVG stains collagen fibers, although fairly) can be used for the visualization of elastic fibers in tissue, while Azan staining helps in the distinction between collagen and reticulin fibers [68,86,95]. In PSR, a possible differentiation in the size of fibers is discussed, while along with MT, it can be also used to evaluate collagen bundle orientation [46,86,89,90]. However, for the most accurate results in collagen birefringence-enhancing PSR, a circularly polarized microscopy should be used [89,90]. MT and PSR stains are characterized by high contrast, which makes them useful in morphometry, microdensitometry, and automatized approaches, but only in colorimetric assessment, as achieved color does not differ considerably in greyscale [86,88,96,97,98]. For basal membrane visualization, Azan or PAS can be used [68,86,94]. Additionally, the Alcian blue stain in varying pH can be used singularly or along with PAS to visualize mucopolysaccharides, as it is specific to carbohydrates and also those bound with proteins [86,92].

The MBABF provides high contrast between cells and ECM, although it is used in specially fixed, epoxy-embedded samples. It is used for the selection of areas of interest for electron microscopy [99,100]. Then, smaller samples are stained with UALC, which non-specifically binds with cellular structures, increases electron density, and thus improves contrast in transmission electron microscopy [87,94,101]. While it is especially useful in cellular structures and degeneration assessment, it is not informative regarding tissue. While most stains include toxic compounds, UALC should be used especially safely [101,102]. An improvement in image quality in scanning electron microscopy can be achieved with gold coating, e.g., with gold sputter coating (GS) [103,104]. While it prevents some artifacts, the scanned surface may appear granular. However, this effect may be reduced with palladium addition [104]. 

The highest specificity of staining can be achieved using the IHC method, as a selected marker is stained with a specific antibody. It allows for highly valuable quantitative analysis, as well as exact antigen localization in the cell and tissue [86,87,105]. Multiple antigens can be visualized in immunostaining when different chromogens or fluorochromes are used [106]. On the other hand, especially in horses, antibodies of proven specificity may not be available and antibody development is expensive, increasing already high costs [87,107]. Tissue processing and antibody binding specificity should undergo an optimization process to achieve the best results. Also, to prove staining specificity, positive and negative controls should be encompassed in the study [87]. Despite the evident value provided by IHC, light microscopy is still a dominant tool in histological specimen evaluation [45]. The properties of stains used in studies concerning connective tissue in the equine cervix, endometrial stroma, and blood vessels are summarized in Table 1, Table 2 and Table 3, as well as compared in Table 4.

## 4. Discussion

It appears that all uterine diseases should be collectively considered in the assessment of expected fertility. Uterine diseases may not occur singularly, which influences their impact on fertility. Conversely, the impact of a similar microscopic picture may vary among individuals, depending on baseline capabilities and adaptability, as overall results depend on a plethora of biological processes and factors [1,6,18,37,116]. Although the co-occurrence of histopathological changes was described in a large sample group, the study was retrospective, and its impact on fertility prognosis was not fully evaluated [72]. Therefore, also in the course of equine endometrosis, the diagnostic description should include more thorough information than just the numerical categorization. Since the first guidelines for the assessment of endometrial changes many studies have been conducted [3,5,28,38], more descriptive results would improve communication between diagnosticians and clinicians, leading to a more accurate diagnosis and decision regarding fertility [5,39]. Only a comprehensive view of both histopathologic and clinical evaluations allows for a reliable assessment of fertility status. On the other hand, a broader description of the reproductive status from which the biopsy was taken would facilitate a more accurate microscopic assessment [1].

While the inadequacy of the Kenney–Doig classification has been previously discussed, this is another report regarding the need for a broader perspective in fertility estimation [1,5,6,37,39]. Although endometrial biopsy is considered the gold standard for diagnosing uterine diseases, there is no gold standard for qualitative evaluation of clinical outcomes and quantification of endometrial fibrosis. The Kenney–Doig categories assigned to the endometrial sample depend clearly on present alterations, but an observer influence was also found, especially since there is no uniform training for assessment and it depends on subjective observations [1,37,117]. Interestingly, two intermediate categories (IIA and IIB) are most frequently found, regardless of age-based division. It is possibly associated with hesitation in classifying the endometrium into extreme categories, describing it as either healthy or hardly able to maintain a pregnancy. In particular, samples varying in the severity of changes along the microscopic slide pose challenges in assessment and classification [1,39,117]. Westendorf et al. (2022) found that one in ten samples evaluated by eight pathologists was classified in all available categories, while even more were assigned to categories I and III by at least two diagnosticians [117]. This general inter- and intra-rater agreement of classification was lower than usually expected in histopathology grading systems. Such inconsistency raises concerns about the reliability of endometrial grading and its repeatability. Thus, despite being considered standardized, the Kenney–Doig classification is subjective and depends on the experience of the pathologist, as well as sampling and tissue characteristics [1,117]. As morphometrics is considered a valuable addition to biopsy assessment, specific staining for connective tissue evaluation may be used for easier differentiation between endometrial compounds, as well as accurate counting of collagen layers encircling a gland [5]. Even if periglandular fibrosis is not the only important alteration in the endometrium, specific staining may help in the detection and quantification of destructive changes in the glandular epithelium [56] and angioses [4,27,35,53,54,55,58,63].

While the benefits from the use of specific staining have clearly been demonstrated, we encourage scientists and diagnosticians to employ any additional tool to improve the visualization of histopathological changes and allow for a more reliable diagnosis regarding expected fertility. As a perspective for further research, the role of microRNAs in the pathogenesis of endometrosis is studied with next-generation sequencing (NGS) [118]. At the same time, blood markers of endometrial fibrosis [23,119], such as collagen type III, have already been investigated. Although it can be suspected that their development and implementation in clinical usage will not occur rapidly, more accessible methods should currently be employed in routine diagnosis. However, regardless of the development and employment of novel and more advanced techniques, light microscopy remains an essential tool in diagnosis and research, complementing newer methods [45]. Especially in the context of endometrosis, in which the main change is increased collagen content, the fact that HE is often the only stain used for biopsy is slightly incomprehensible. MT may be helpful in the connective tissue evaluation and quantification both in the cervical and uterine mucous membrane stroma as well as in the blood vessel wall.

## 5. Conclusions

Several methods for connective tissue staining in mares’ uterus and cervix are available. Among them, MT and PSR stains provide high contrast of staining, useful for quantitative fibrosis evaluation; GT is simple, yet highly reliable; EVG differentiates types of connective tissue fibers; and PAS effectively stains basal membranes and neutral mucins. Therefore, regarding assessment of the connective tissue and fibrosis, each of them represents benefits over conventional HE, although selection should be made based on the purpose and aim of a study or diagnosis. The clinical impact of the use of specific staining includes improved visualization of the connective tissue, which may enhance the accurate assessment of fibrosis-related diseases of the equine endometrium. However, their use in the quantification of endometrial fibrosis requires further research linked with the qualitative evaluation of clinical outcomes and fertility.

## Figures and Tables

**Table 1:** Histological assessment of the structure and function of the cervix.

Assessments	Demographics	Specimen	Staining	Results	Ref.
1. Cervical collagen content concerning the phase of the ovarian cycle.	29 non-pregnant mares; Age not reported	Full-thickness post-mortem cervical sections ^a,b^	HE; MT	1. The collagen in the equine cervix undergoes hormonal regulation; 2. The collagen content was higher in tunica mucosa in estrus than diestrus.	[50]
1. Cervical proportion of collagen content and estrogen receptor concerning the phase of the ovarian cycle.	12 non-pregnant mares; Age not reported	Full-thickness post-mortem cervical sections ^a^	MT; IHC	1. Expression of estrogen receptor α in cervical wall was higher in estrus than diestrus; 2. Expression of estrogen receptor α was negatively correlated with collagen content.	[51]
1. Cervical function based on the histological findings.	10 non-pregnant mares; 7–25 years	Full-thickness post-mortem cervical sections ^a^	HE; PAS; Azan; RF	1. Ciliated cells form luminal epithelium; 2. The deep lamina propria of mucosae show high vascularization.	[65]
1. Inflammatory and endocrine factors (mRNA expression) in relation to the histological findings.	9 pregnant mares; 4–9 years	Full-thickness post-mortem cervical sections ^a^	HE	1. Cervicitis is characterized by epithelial erosion and loss of tissue architecture; 2. Cervicitis is characterized by epithelial cell necrosis and desquamation	[73]

Footnotes: specimen assessed in the following: ^a^ light microscopy; ^b^ scanning cytometer; HE, hematoxylin–eosin staining; PAS, periodic acid–Schiff staining; RF, resorcin–fuchsin staining; MT, Masson trichrome staining; IHC, immunohistochemical staining. Ref., references.

**Table 2:** Histological assessment of uterine vascular changes.

Assessments	Demographics	Specimen	Staining	Results	Ref.
1. Distribution pattern of the characteristic lesions of endometrosis.	50 non-pregnant mares; 1–30 years	Full-thickness post-mortem uterine sections ^a,b^	HE; EVG; PAS; UALC	1. Vascular lesions first appeared in 6-year-old mares, then increases in both severity and incidence with age; 2. Angiosclerosis incidence and degree increase with age, not parity.	[4]
1. Detailed histomorphological characterization of endometrial biopsies of old mares.	819 non-pregnant mares; Age > 20 years	Endometrial biopsies ^a^	HE PSR	1. Only in 8% of samples angiosclerosis was not found in HE, however, PSR showed alterations in most of them.	[27]
1. Endometrial angiopathies and elastic fibers; 2. Effect of ageing and parturition on angiopathies.	117 non-pregnant mares; Age not reported	Endometrial biopsies ^a,b^	HE; PSR; MBABF; UALC	1. Mild intimal and perivascular sclerosis was age-related; 2. Angiosis increased in frequency with the number of foalings; 3. Ageing and degeneration of elastic fibers facilitates elastase activity.	[35]
1. Occurrence of endometrial alterations in age groups.	9121 non-pregnant mares; 1–30 years	Endometrial biopsies ^a^	HE *	1. Incidence and degree of angiosis increase with mare age.	[40]
1. Angiopathies in the course of endometritis and endometrosis.	14 non-pregnant mares; Age not reported	Full-thickness post-mortem uterine sections ^a,c^	HE; MT	1. Wall thinning vasodilation was found in endometritis and endometrosis; 2. Vasodilation was lower in endometritis than endometrosis.	[53]
1. Angiopathies of different endometrosis severity.	24 non-pregnant mares; Age not reported	Full-thickness post-mortem uterine sections ^a,c^	HE; MT	1. Incidence and degree of angiopathies increase with endometrosis severity; 2. Perivascular fibrosis, wall thinning, and vasodilation occurs in severe endometrosis.	[54]
1. Angiopathies and hyaluronan synthases expression of different endometrosis severity.	24 non-pregnant mares; Age not reported	Full-thickness post-mortem uterine sections ^a,c^	HE; MT	1. Perivascular fibrosis in endometrium affected by severe endometrosis may be mediated by hyaluronan synthases 1 and 3.	[55]
1. Detection of β-defensin the healthy and diseased endometrium; 2. Characterization of the cell population(s) expressing β-defensin.	11 non-pregnant mares (post-mortem sections); 18 non-pregnant mares, (endometrial biopsies)	Full-thickness post-mortem uterine sections; Endometrial biopsies ^a^	HE PSR	1. β-defensin was found in luminal and glandular epithelium, tunica media of endometrial vessels, vascular smooth muscle cells.	[58]
1. Evaluation of relationship between the appearance of small arteries under endometrium (SAUE) in histology and endoscopy.	7 mares (post-mortem sections); 423 non-pregnant mares, aged 4–20 years (endometrial biopsies)	Full-thickness post-mortem uterine sections; Endometrial biopsies ^a^	HE EVG	1. Endoscopic appearance of SAUE reflects the sclerotic change in the intima and adventitia; 2. Small arteries in the endometrium show age-related sclerotic changes: elastosis in the intima and adventitia, resulting in luminal narrowing.	[63]
1. Uterine blood flow of different ages and endometrosis severity in the first 20 days of gestation.	21 pregnant mares; 4–18 years	Endometrial biopsies ^a^	HE	1. Uterine blood flow increase with gestational age regardless of age end endometrial changes; 2. Uterine blood flow differed according to the embryo position which is less pronounced in older mares and mares with endometrosis.	[77]

Footnotes: specimen assessed in the following: ^a^ light microscopy; ^b^ electron microscopy; ^c^ scanning cytometer; HE, hematoxylin–eosin staining; PSR, picrosirius red staining; MBABF, methylene-blue-azur2-basic fuchsin staining; UALC, uranyl acetate–lead citrate staining; EVG, elastica van Gieson staining; PAS, periodic acid–Schiff staining; MT, Masson trichrome staining. * staining not directly reported, presumably HE; Ref., references.

**Table 3:** Histological assessment of endometrial changes and their impact on fertility.

Assessments	Demographics	Specimen	Staining	Results	Ref.
1. Distribution pattern of the characteristic lesions of endometrosis.	50 non-pregnant mares; 1–30 years	Full-thickness post-mortem uterine sections ^a,b^	HE; EVG; PAS; UALC	1. Occurrence of endometrosis increase with mare age.	[4]
1. Subfertility of retired sports mares.	189 non-pregnant sports mares; 3–23 years	Endometrial biopsies ^a^	HE; IHC	1. Higher glandular differentiation disorders in retired sport mares than non-performance mares;	[6]
1. Periglandular fibrosis (quantification) in the course of endometrosis.	70 non-pregnant mares; 3–27 years	Endometrial biopsies ^a^	HE; PSR	1. Periglandular fibrosis (collagen volume fraction) correlates with endometrosis severity.	[15]
1. Precise detection of fibrotic changes in the course of endometrosis.	40 non-pregnant; 5–18 years	Endometrial biopsies ^a^	HE; MT; AB; IHC	1. Combination of different staining methods is useful for endometrosis assessment.	[16]
1. Collagen deposits and metalloproteinases activity in the course of endometrosis.	44 non-pregnant mares; 5–27 years	Endometrial biopsies ^a^	HE; PSR	1. No correlation between infertility, endometrosis severity, amount of collagen and metalloproteinases activity.	[18]
1. Fibrosis and glandular nests in the course of endometrosis.	377 non-pregnant mares; 16.41 ± 0.41 years	Endometrial biopsies ^a^	HE; MT	1. Fibrosis intensity and glandular nests occurrence increase with endometrosis severity.	[22]
1. Detailed histomorphological characterization of endometrial biopsies of old mares.	819 non-pregnant mares; Age > 20 years	Endometrial biopsies ^a^	HE	1. Endometrosis was found in 97% of samples, mostly with periglandular inflammatory cells (58%).	[27]
1. Occurrence of endometrial alterations in age groups.	9121 non-pregnant mares; 1–30 years	Endometrial biopsies ^a^	HE *	1. Incidence of endometrosis increase with mare age.	[40]
1. Fibrosis in endometrial samples.	5 non-pregnant mares; Age not reported	Full-thickness post-mortem uterine sections ^a^	HE; MT	1. No differences in HE and MT assessment of periglandular fibrosis.	[43]
1. Evaluation of endometrosis features; 2. Evaluation of mucopolysaccharides in ECM.	25 multiparous mares; 7–19 years	Endometrial biopsies ^a^	HE; MT; PAS; AB	1. Defensin-β 4B may be involved in regulation of immune response and indirectly influence ECM formation.	[52]
1. Color of cytoplasm and fibrosis in the course of endometrosis.	20 non-pregnant mares; Age not reported	Full-thickness post-mortem uterine sections ^a,c^	HE; MT	1. Light cytoplasm area in glands increase with endometrosis severity; 2. Fibrotic area was larger around light than dark cytoplasm glands.	[56]
1. Differences in cytoplasm staining between normal and degenerated glands in the course of endometrosis.	20 non-pregnant mares; Age not reported	Full-thickness post-mortem uterine sections ^a,c^	HE; MT	1. Percentage of light cytoplasm glands is higher in severe endometrosis than in healthy endometrium; 2. No differences in dark cytoplasm glands concerning endometrosis severity.	[57]
1. Determination of the effects of kerosene on endometrium; 2. Assessment of hysteroscopy and transrectal ultrasound to detect the presence of active endometrial cups.	9 mares (pregnant, terminated during study	Endometrial biopsies ^a^	HE; PSR; IHC	1. Intrauterine kerosene infusions do not hasten regression of retained endometrial cups following an abortion; 2. Intrauterine kerosene infusions in mares did not appear to affect mare health or endometrium.	[60]
1. Evaluation of endometritis and chronic fibrosis after enrofloxacin infusion administration	9 non-pregnant mares 5–13 years	Endometrial biopsies ^a^	HE GT	1. Infusion of enrofloxacin for treatment of endometritis induced severe acute endometrial mucosal necrosis and significant chronic endometrial fibrosis and inflammation	[61]
1. Evaluation of endometrosis features; 2. Evaluation of placental surface.	9 pregnant mares; Healthy group: 4–12 years; Endometrosis group: 10–22 years	Samples from allantochorion and endometrium ^a,b^; Endometrial biopsies ^a^; Both post-mortem	HE; EVG; UALC; GS coating	1. The poorest development of placenta and microcotyledons was found in association with glandular atrophy; 2. Pregnancy-induced increase in the density of endometrial glands.	[64]
1. Evaluation of the ultrastructural and histological changes in the endometrium in days 7, 10, and 13 post-ovulation in pregnant and cyclic mares	30 mares; 5–10 years	Endometrial biopsies ^a,b^	HE PAS GS coating	1. In the stroma and lumen, modifications occurred to provide nutrition necessary for the embryo and to promote changes that will interact in the embryonic signaling and future fixation, implantation, and placentation.	[66]
1. Evaluation of presence and distribution of ECM proteins; 2. Histochemical characteristics of fibroblasts.	50 non-pregnant mares; 5–23 years	Endometrial biopsies ^a^	HE; IHC	1. Endometrotic tissue is characterized by periglandularly arranged fibroblasts producing collagen IV, laminin, and fibronectin; 2. Fibroblasts express α-smooth muscle actin, tropomyosin, and occasionally desmin.	[67]

Footnotes: specimen assessed in the following: ^a^ light microscopy; ^b^ electron microscopy; ^c^ scanning cytometer; HE, hematoxylin–eosin staining; MT, Masson trichrome staining; PSR, picrosirius red staining; EVG, elastica van Gieson staining; PAS, periodic acid–Schiff staining; UALC, uranyl acetate–lead citrate staining; IHC, immunohistochemical staining; AB, Alcian blue staining; GS, gold sputter coating. * Staining not directly reported, presumably HE; Ref., references.

**Table 4:** Comparison of staining methods employed for the histological assessment of the connective tissue previously.

Staining	Purpose of Staining	Specimen	Advantages	Disadvantages
HE	Visualization of cellular morphology and tissue architecture [45,85]	Full-thickness cervical sections [50,51,65,73]; Endometrial biopsies [6,15,16,18,35,40,52,55,58,60,61,63,64,66,77]; Full-thickness uterine sections [4,53,54,55,56,57,58,63]	Quick, easy, cheap Versatile [85,86].	Is not selective to connective tissue fibers; Only for visual analysis [69,87,88].
MT	Visualization of collagen, elastic fibers, muscle, and epithelium [108]	Full-thickness cervical sections [50,51]; Endometrial biopsies [52]; Full-thickness uterine sections [43,53,54,55,56,57]	High contrast between stained structures; Stains all types of collagen fibers and elastic fibers [85,86]; Allows for microdensitometry [96].	Some thin fibers may not be stained; Lack of differentiation between types of fibers; Corrosive reagents used [86,87].
PSR	Visualization of collagen [85,86]	Endometrial biopsies [15,18,35,58,59,60]; Full-thickness uterine sections [58]	Stable, fast, consistent, inexpensive; Highest contrast among other stains [89]; Stains basal lamina; Allows for microdensitometry [86,88].	Polarized microscope recommended; Rotation of the slide; Poorer staining of nuclei [88]; Requires understanding of polarization effects [86,89].
GT	Visualization of muscles and connective tissue [108,109]	Endometrial biopsies [61,62]	Stable reagents; Simple, one-step staining; Highly reliable [47].	Overstaining when ethanol-fixed samples are used [92].
EVG	Visualization of elastic tissue; Visualization of collagen and enhancing nuclear detail [95,108]	Endometrial biopsies [63,64]; Full-thickness uterine sections [4,63]	Large numbers of sections can be easily stained by van Gieson’s method and its modifications; Good visualization of all types of fibers [86]; Highest contrast of elastic fibers among other stains [93].	Non-detailed cytoplasm; Fading depending on the mounting medium; Variability in acid fuchsin quality [86]; Poor collagen staining [91]; High variability in differentiation, slides should be evaluated separately [93].
Azan	Visualization of collagen, reticulin fibers, basal membrane [86]	Full-thickness cervical sections [65]	High control of the HE intensity [86].	Time consuming and troublesome; Variable differentiation [86,94].
RF	Elastic fibers staining [110]	Full-thickness cervical sections [65]	Reliable, good contrast of elastic fibers [93].	Not fully specific [111]; Variable differentiation [110]; Staining and reagent preparation is time-consuming [93].
PAS	Identification of glycogen, neutral mucins, basement membrane, collagen fibers [94,108]	Full-thickness cervical sections [65]; Endometrial biopsies [52,66]; Full-thickness uterine sections [4]	Good for basal membrane staining [94,112]; Visualizes reticulin fibers [112]; Combined with alcian blue stains mucopolysaccharides [86].	Weak staining of collagen; Cannot be used singlehandedly to evaluate connective tissue [112].
MBABF	Visualization of connective tissue and muscles [100]; Selection of sites for electron microscopy [113]	Endometrial biopsies [35]	High contrast; Enables morphometry [99].	Variable staining intensity; Semi-thin slices only [113]
UALC (electron microscopy)	Contrast for cellular structures evaluation in transmission electron microscopy [87,94,101]	Endometrial biopsies [35,64]; Full-thickness uterine sections [4]	Versatile; High contrast [114].	Unpredictable; Sensitive to light; Highly toxic; Restricted use [87,94,101,102].
GS coating (electron microscopy)	Providing a homogeneous surface for analysis and imaging in scanning electron microscope; Preventing charging of the surface [103]	Samples from allantochorion and endometrium [64]; Endometrial biopsies [66]	Non-oxidizing, excellent conductor; Reduces beam-penetration artifacts [115].	Granular and cracked appearance of the surface (reduced by palladium addition) [104].
IHC	Expression and localization of selected marker [86,87]	Full-thickness cervical sections [51]; Endometrial biopsies [6,16,60,67]	High specificity Direct localization of an antigen [86]; Wide range of applications [87].	Requires optimization and controls. Requires tissue processing; Expensive availability of specific antibodies [87].

Footnotes: HE, hematoxylin–eosin staining; MT, Masson trichrome staining; PSR, picrosirius red staining; GT, Gomori trichrome staining; EVG, elastica van Gieson staining; RF, resorcin–fuchsin staining; PAS, periodic acid–Schiff staining; MBABF, methylene-blue-azur2-basic fuchsin staining; UALC, uranyl acetate–lead citrate staining; GS, gold sputter coating; IHC, immunohistochemical staining.

## Data Availability

The data presented in this study are available on request from the corresponding author.
